# Age-related visual impairments and retinal ganglion cells axonal degeneration in a mouse model harboring OPTN (E50K) mutation

**DOI:** 10.1038/s41419-022-04836-3

**Published:** 2022-04-18

**Authors:** Mingying Hou, Zhengbo Shao, Shiqi Zhang, Xinna Liu, Pan Fan, Menglu Jiang, Yutong Zhao, Rong Xiao, Huiping Yuan

**Affiliations:** 1 Department of Ophthalmology, The Second Affiliated Hospital of Harbin Medical University, Harbin, China; 2 The Key Laboratory of Myocardial Ischemia, Harbin Medical University, Ministry of Education, Harbin, China; 3 Future Medical Laboratory, The Second Affiliated Hospital of Harbin Medical University, Harbin, China

**Keywords:** Neuroscience, Medical research

## Abstract

Retinal ganglion cells (RGCs) axons are the signal carriers of visual information between retina and brain. Therefore, they play one of the important roles affected in many optic neurodegenerative diseases like glaucoma. Among the genetic risks associated with glaucoma, the E50K mutation in the Optineurin (OPTN) gene are known to result in glaucoma in the absence of increased intraocular pressure (IOP), whereas the relevant pathological mechanism and neurological issues remain to be further investigated. In this study, the OPTN (E50K) mutant mouse model was established through CRISPR/Cas9-mediated genome editing, and aging-related RGCs loss and the visual dysfunction were identified. In E50K mice 16 months old, the axonal transport decreased comparing to wild-type (WT) mice at the same age. Furthermore, results of electron microscopy demonstrated significant morphological anomaly of mitochondria in RGCs axons of young E50K mice 3 months old, and these changes were aggravated with age. These indicated that the damaged mitochondria-associated dysfunction of RGCs axon should play an etiological role in glaucoma as an age-related outcome of OPTN (E50K) mutation. The findings of this study have potential implications for the targeted prevention and treatment of NTG.

## Introduction

Glaucoma, a group of eye diseases causing damage to optic nerve, is the most common cause of irreversible blindness and visual impairments worldwide [1]. Damage to the optic nerve may occur with or without an increase in IOP. In the latter cases, glaucoma often continues to progress even with a reduced IOP regimen, and eventually develops irreversible visual loss [2]. A study based on clinical trial found no evidence of increased trans-lamina cribrosa pressure difference (TLCPD) or reduced intracranial pressure (ICP) as the major pathogenesis in normal tension glaucoma (NTG) [3], a subtype of primary open-angle glaucoma (POAG), thus suggesting that pressure-independent rick factors may play an essential role in its etiology. In 2002, a gene involved in inherited NTG was identified and renamed after Optineurin (OPTN) [4], and its genetic mutation may cause adult-onset POAG. This discovery gave a perspective to explore the causes and develop the control strategy for such diseases. In the later studies, among the identified OPTN mutant patterns, E50K is particularly associated with familial glaucoma without IOP increase [5], and the relevant clinical manifestations are more severe and the onset of NTG in the E50K mutant appeared earlier [6, 7]. The evolvement into visual impairments may be related to age, and their pathogenesis needs to be further explored.

In glaucoma development, the RGCs soma and their axons, or even the related brain tissues may be involved in the neurodegeneration [8, 9]. RGCs axons connect the retina to the brain, mainly performing both visual signal transmission and axonal transport. In anterograde axonal transport, vesicles formed in RGCs soma are actively transported along axonal microtubules to presynaptic site. In retrograde axonal transport, neurotrophic factors and degradative vesicles carrying aged organelles or aggregated proteins take the opposite route. Maintenance of RGCs and their axons is dependent upon these bio-processes [10]. Failure of them may be an early sign of RGCs degeneration. Previous studies revealed that disorders of axonal transport were closely associated with clinical symptoms of Alzheimer’s disease, Parkinson’s disease and Huntington’s disease [11]. In some animal models of chronic glaucoma with high IOP, RGCs axonal damage was detected earlier than neuron loss [12]. These studies indicated that axonal degeneration could be the cause of a variety of neurodegenerative diseases like glaucoma, rather than the result of disease development.

In this study, a mouse model harboring OPTN (E50K) mutation was established through CRISPR/Cas9-mediated genome editing. Even though the reduced thickness of the RGCs distributing layers in retina and the loss of the viable RGCs progressed with age in wild type (WT) mice, this age-related degeneration got even worse in E50K mice. Additionally, the significant visual impairments and decreased retrograde and anterograde axonal transport were detected in E50K mice aged 16 months (E50K mice-16M). We then found that the damage of RGCs axonal mitochondria in E50K mice-3M precedes the degeneration of the RGCs axon in E50K mice-16M. These results suggested that RGCs axonal degeneration should work in the pathogenesis of visual impairments induced by OPTN (E50K) mutation, and accumulated mitochondrial damage from young age might play an significant role etiologically. These findings provide a new perspective for the selection of new treatment strategies and targets for NTG.

## Results

### Establishment and phenotypic validation of OPTN (E50K) mutant homozygous mouse

**Fig. 1:**
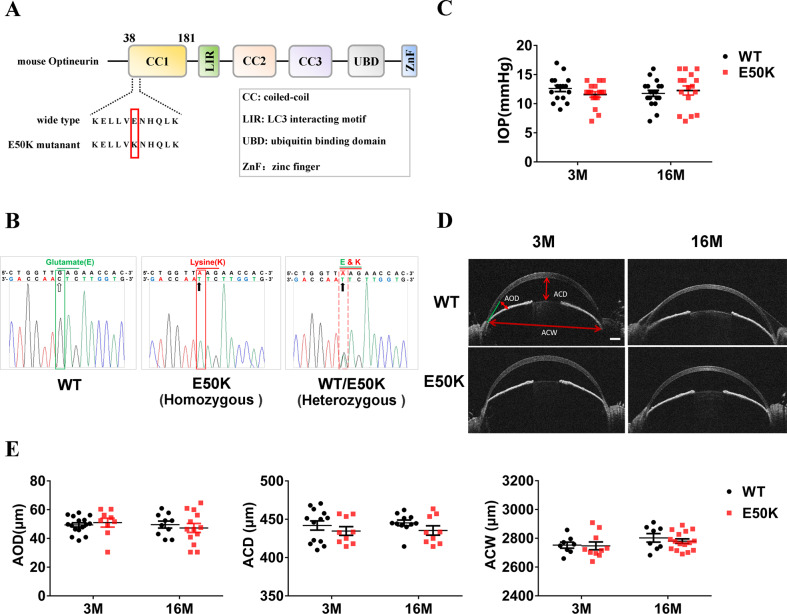
Generation and identification of OPTN (E50K) mutant mice model. **A** Generation of OPTN (E50K) mutant homozygote of mice based on CRISPR/Cas9-mediated genome editing. **B** Based on PCR product sequencing, homozygous mutant mice were identified and used as E50K mice in this study. **C** The IOP in mice eyes based on measurement of a rebound tonometer (*P* > 0.05, *n* = 16–18). **D**, **E** The anterior chamber structures of mice were imaged through AS-OCT, and three parameters, ACD (the distance from the posterior cornea surface to the anterior surface of the lens), AOD (the distance between the posterior cornea and anterior iris surface, along a line perpendicular to the cornea at 500 μm from the point of iridoscleral contact), and ACW (the distance between the two iridoscleral contacts) were analyzed. Scale bar in **D**, 250 μm. (*P* > 0.05, *n* = 8–16).

E50K mutant mice constructed by CRISPR/Cas9 gene-editing technology were identified by PCR product sequencing, and the mutant homozygous mice were selected for phenotypic verification (Fig. 1A, B). Intraocular pressure (IOP) and anterior chamber structures were measured by the rebound tonometer and anterior segment optical coherence tomography (AS-OCT), respectively. The results showed no significant difference in IOP between WT mice and E50K mice at the age of 3 months and 16 months (*P* > 0.05, *n* = 16–18) (Fig. 1C). No significant difference between WT mice and E50K mice was found in anterior chamber depth (ACD), anterior chamber width (ACW) and angle opening distance (AOD), the three main parameters reflecting anterior chamber structures (*P* > 0.05, *n* = 8–16) (Fig. 1D, E). These results indicated that the OPTN (E50K) mutant mice model constructed in this study does not change IOP and anterior chamber structure, which is consistent with the clinical NTG phenotype [7].

**Fig. 2:**
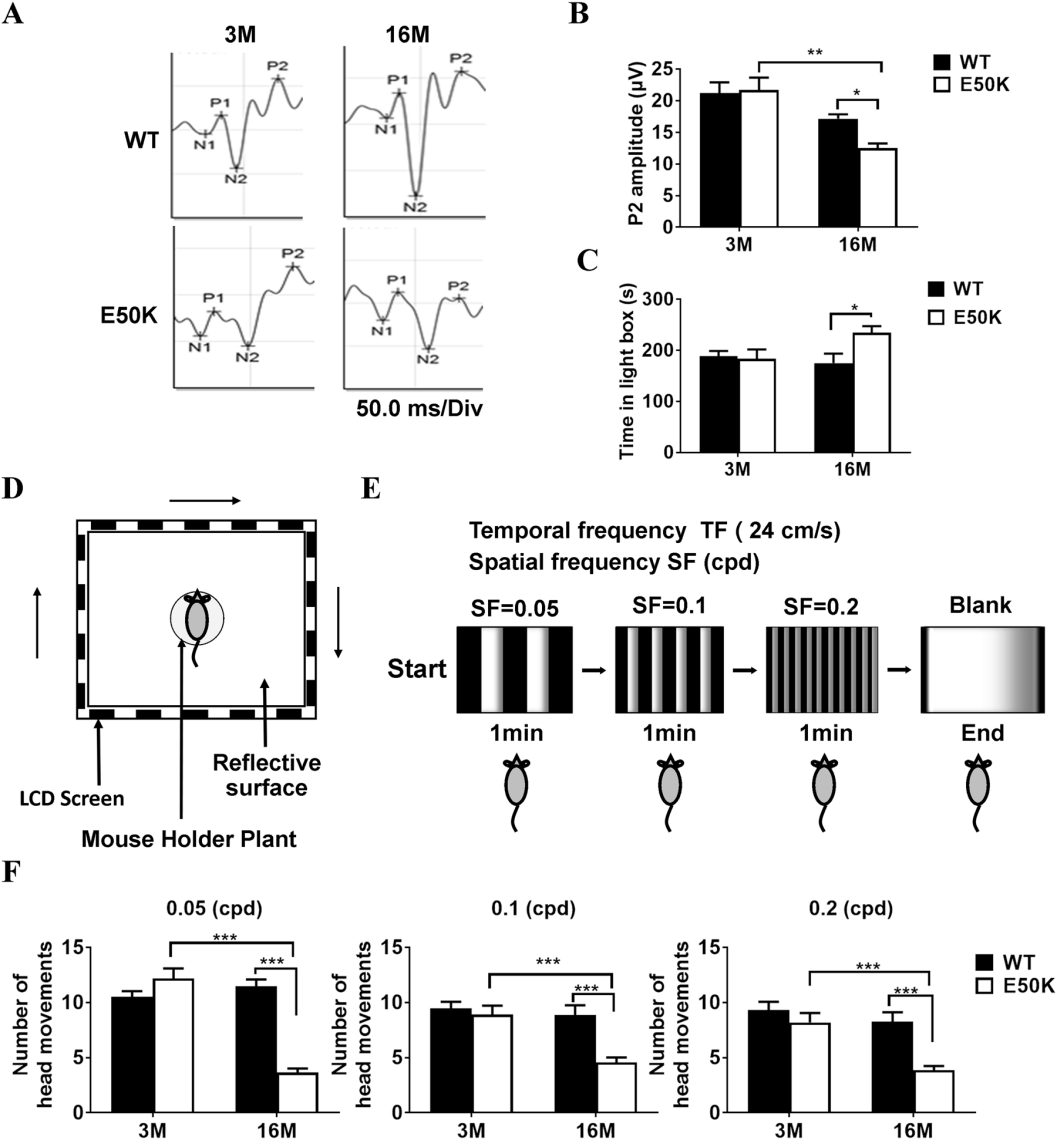
Evaluation of visual function in WT and OPTN (E50K) mice. **A**, **B** F-VEP was used to access the light reflex in WT and E50K mice. (WT-3M, 21.2 ± 1.6, *n* = 31, E50K-3M, 21.6 ± 1.9, *n* = 21, WT-16M, 17.1 ± 0.7, *n* = 41, E50K-16M, 12.4 ± 0.7, *n* = 43). **C** With a designed Light/Dark Transition apparatus, the length of time the free-roaming mice spent in light space was monitored. (WT-3M, 188.8 ± 9.9, *n* = 17, E50K-3M, 183.6 ± 18.3, *n* = 10, WT-16M, 175.2 ± 18.3, *n* = 22,E50K-16M, 234.4 ± 12.9, *n* = 31). **D**–**F** With a designed OMR test apparatus, the head movement was captured to indicate the response to object movement at different frequencies in the environment. The top-view photograph of the OMR system consisted of a visual stimulus unit, which displays clockwise rotation of black-and-white stripes on 4 LCD screens (**D**, **E**). The number of OMR under each condition was analyzed (**F**). ①0.05 cpd: WT-3M, 10.5 ± 0.5, *n* = 36, E50K-3M, 12.1 ± 0.9, *n* = 11, WT-16M, 11.4 ± 0.62, *n* = 13, E50K-16M, 3.6 ± 0.4, *n* = 16. ②0.1 cpd: WT-3M, 9.4 ± 0.5, *n* = 36, E50K-3M, 8.9 ± 0.8, *n* = 13, WT-16M, 8.8 ± 0.8, *n* = 19, E50K-16M, 4.5 ± 0.4, *n* = 16. ③0.2 cpd: WT-3M, 9.3 ± 0.7, *n* = 24, E50K-3M, 8.2 ± 0.8, *n* = 10, WT-16M, 8.2 ± 0.8, *n* = 17, E50K-16M, 3.8 ± 0.3, *n* = 14. (*P < 0.05, **P < 0.01, ***P < 0.001).

The results of Flash visual-evoked potentials (F-VEP) demonstrated that the P2-wave amplitude detected in E50K mice-16M was significantly lower than that in WT mice-16M (12.5 ± 0.7, *n* = 43 vs. 17.1 ± 0.7, *n* = 41, *P* < 0.05) (Fig. 2A, B). The E50K mice-16M also spent more time than WT mice-16M (234.4 ± 12.9, *n* = 31 vs. 175.2 ± 18.3, *n* = 22, *P* < 0.05) in the light environment in Light/Dark transition (L/D-T) test (Fig. 2C), suggesting reduced visual sensitivity to light. In addition, Optomotor response (OMR) test was performed after light adaptation in mice (Fig. 2D, E). The head movement times of E50K mice-16M were significantly lower than those of WT mice and E50K mice-3M at frequencies of 0.05 cpd (cycle per degree), 0.1 cpd and 0.2 cpd (*P* < 0.001) (Fig. 2F). According to these findings, different from E50K mice-16M, both WT mice-3M & 16M and E50K mice-3M could perceive the brightness of light, like those used in F-VEP and L/D-T, and respond to more complex visual stimulation, such as that used to measure the OMR. These results above suggested that the visual impairment phenotype induced by E50K mutation in mice was gradually aggravated with age.

### Expression of OPTN in retina of E50K mice

**Fig. 3:**
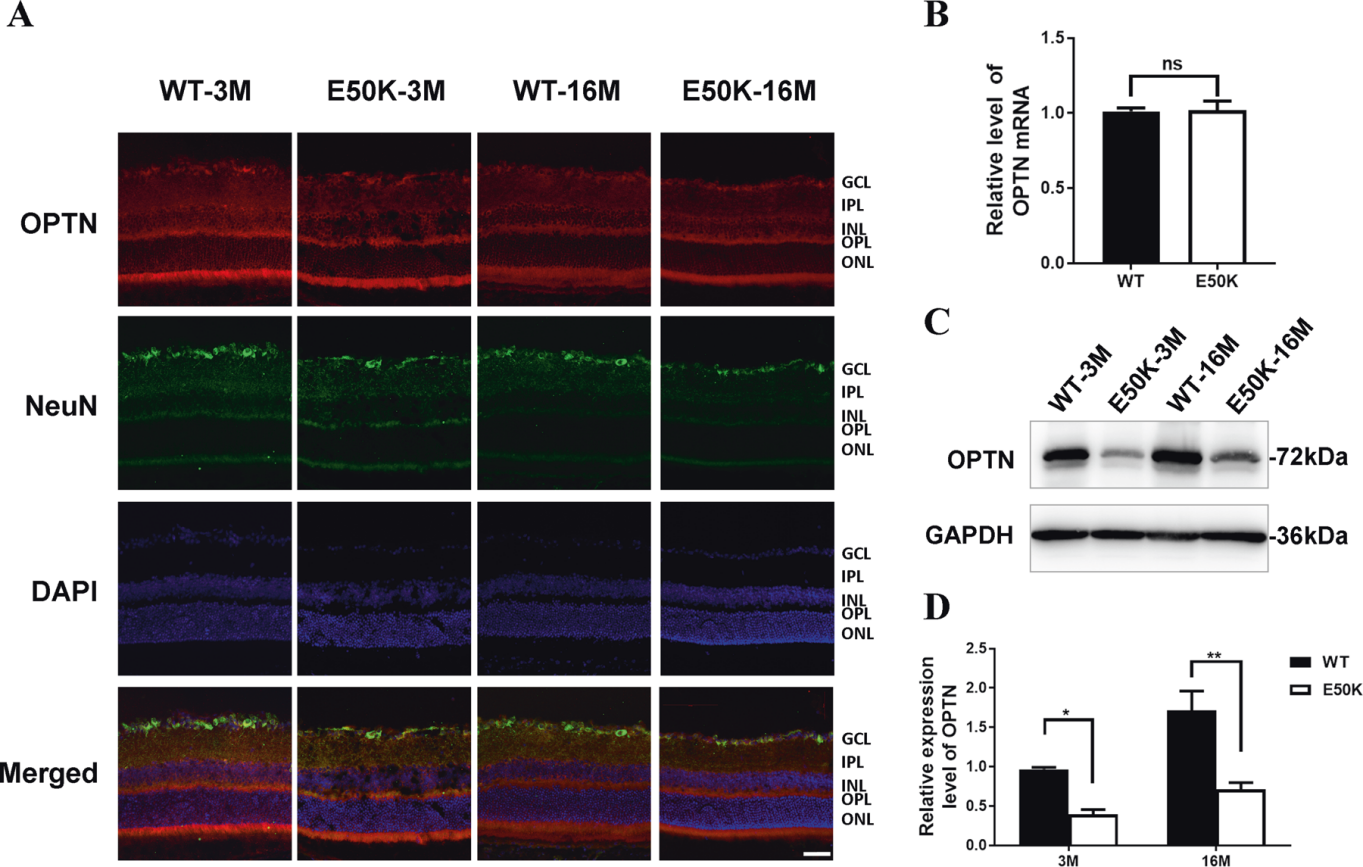
OPTN expression in retina of WT and OPTN (E50K) mice. **A** After frozen section was prepared, immunohistochemical method was used to mark OPTN protein in red and RGCs in green. DAPI was used to mark cell nuclear in blue. The red color indicated the distribution of OPTN protein in layers of retina. The green color labeled the soma of RGCs in retina. Scale bar, 50 μm. **B** The mRNA level of total OPTN was evaluated by RT-qPCR. Student’s *t*-test. **C**, **D** The protein level of total OPTN was determined by WB. The protein expression was compared by bands gray value. (*P < 0.05, **P < 0.01).

We prepared frozen sections of the eyeballs and used NeuN to mark the RGCs soma distribution. Immunofluorescence results showed that OPTN was expressed in nearly all layers of internal retina, and there was no significant difference between WT and E50K mice at both 3 months and 16 months old (Fig. 3A). These results indicated that E50K mutation did not affect the expression and distribution of OPTN protein in retina.

Total RNA was extracted from retinal tissues of WT mice-3M and E50K mice-3M, and the mRNA expression level of total OPTN was evaluated by RT-qPCR (*P* > 0.05) (Fig. 3B). The results also indicated that the total expression level of OPTN was not affected by E50K mutation. Then, mice retinal tissue was harvested to determine the expression level of OPTN protein by WB (Fig. 3C). The results showed that the level of OPTN protein in the cell lysate supernatant of E50K mice aged 3 months and 16 months was lower than that of WT mice of the same age (Fig. 3D). Combined with the OPTN mRNA expression, the level of soluble OPTN protein in the retina of the E50K mice was lower than that in the WT mice, that is, the total OPTN protein contained insoluble part.

### Changes of retinal structures in E50K mice

**Fig. 4:**
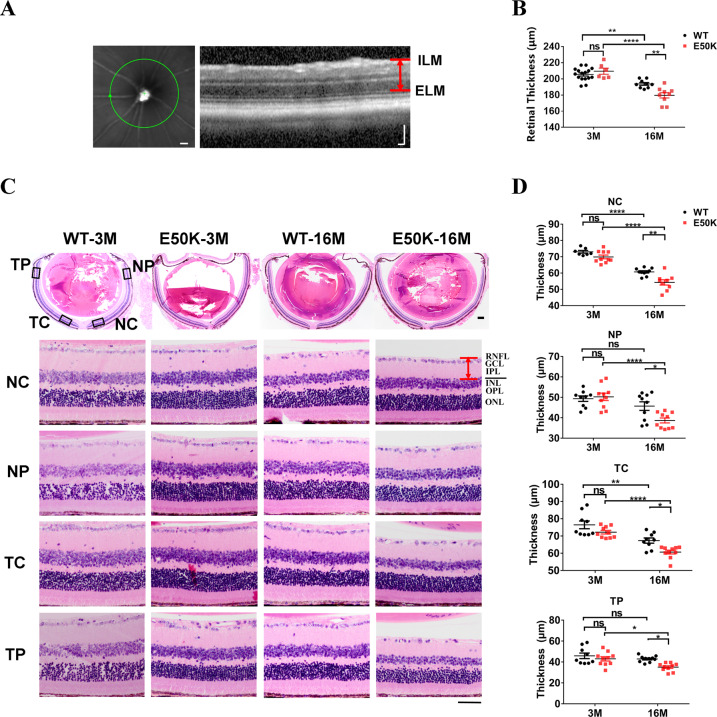
Retina structural changes in WT and OPTN (E50K) mice. **A**, **B** The retinal thickness was measured by PS-OCT. Scale bar in **A**, 500 μm (left panel), 100 μm (right panel). (E50K-16M, 179.7 ± 3.4, *n* = 9, WT-16M, 193.8 ± 1.7, *n* = 9, *P* < 0.01). **C**, **D** The paraffin section of retinal tissue was stained by H&E. According to the previous data, we found the inhomogeneity of retinal thickness. Therefore, four parts of NC (nasal center), NP (nasal periphery), TC (temporal center) and TP (temporal periphery) were grouped to measure the total thickness of retinal-associated layers (RNFL, GCL and IPL) in each retina sample. Scale bars in **C**, 200 μm (upper panel), 50 μm (lower panel). (NC: WT-16M, 60.7 ± 0.7, *n* = 10, E50K-16M, 54.3 ± 1.6, *n* = 9; NP: WT-16M, 45.7 ± 2.1, *n* = 10, E50K-16M, 38.7 ± 1.2, *n* = 10; TC: WT-16M, 67.4 ± 1.5, *n* = 9, E50K-16M, 60.7 ± 1.1, *n* = 10; TP: WT-16M, 42.9 ± 0.9, *n* = 10, E50K-16M, 35.1 ± 1.2, *n* = 10). (**P < 0.01, ***P < 0.001).

Posterior segment optical coherence tomography (PS-OCT) imaging was used to measure the retinal thickness of the mice in each group (Fig. 4A), which found the E50K mice-16M had significantly lower retinal thickness than the WT mice-16M (179.7 ± 3.4, *n* = 9 vs. 193.8 ± 1.7, *n* = 9, *P* < 0.01) (Fig. 4B). In view of the microscopic multi-layer of retina, paraffin sections were prepared, and hematoxylin and eosin (H&E) staining was performed to measure the total thickness of the RGCs distributing layers including retinal nerve fiber layer (RNFL), ganglion cell layer (GCL) and inner plexiform layer (IPL).The retinal thickness from the nasal to the temporal is different, therefore in order to avoid errors caused by random sites, we distinguished the nasal side from the temporal side, and the central area from the peripheral area. No significant difference was found in the thickness of RGCs distributing layers between WT mice and E50K mice at 3 months age (*P* > 0.05, *n* = 7–10). Nevertheless the thickness of RGCs distributing layers was significantly reduced in E50K mice-16M (Fig. 4D). These data revealed that the RGCs distributing layers were significantly degraded in E50K mice, and age played a marked role in RGCs loss, which was confirmed by our results in paraffin sectioning, PS-OCT and visual function tests.

### RGCs loss in retina of E50K mice

**Fig. 5:**
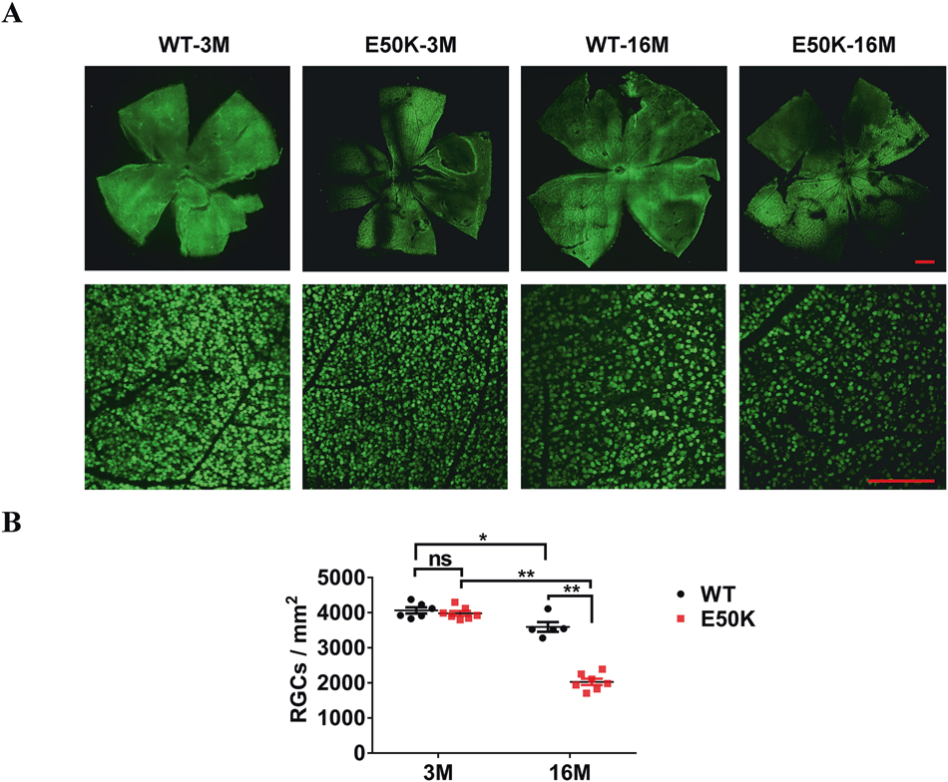
RGCs quantification in WT and OPTN (E50K) mice. **A** Based on flat mount of retina, RGCs was marked by NeuN in green, multiple tiled images were captured and then assembled into a mosaic image of the whole retina flat mount using a confocal fluorescence microscope. Scale bars, 500 μm (upper panel), 200 μm (lower panel). **B** Using ImageJ, the cell density of RGCs labeled in green was calculated (WT-3M, 4065.5 ± 87.5, *n* = 6, E50K-3M, 3982.0 ± 66.1, *n* = 7, WT-16M, 3594.8 ± 137.9, *n* = 5, E50K-16M, 2027.9 ± 90.0, *n* = 7, *P < 0.05, **P < 0.01).

RGCs quantitation was performed with flat mount of retina. (Fig. 5A). The results showed that the densities of RGCs in E50K mice-16M was lower than that in WT mice-16M (2027.9 ± 90.0, *n* = 7 vs. 3594.8 ± 137.9, *n* = 5, *P* < 0.01) and E50K mice-3M (3982 ± 66.1, *n* = 7, *P* < 0.01). Although the density of RGCs in WT mice was also decreased with age (4065.5 ± 87.5, *n* = 6 vs. 3594.8 ± 137.9, *n* = 5, *P* < 0.05), the E50K mice showed more severe RGCs loss with age (3982 ± 66.1, *n* = 7 vs. 2027.9 ± 90.0, *n* = 7, *P* < 0.01) (Fig. 5B). These results suggested that although the decline with age in the density of RGCs was found in WT mice-16M, this age-related degeneration of RGCs was more severe in the retina of E50K mutant mice.

### Impaired axonal transport in optic nerve of E50K mice

**Fig. 6:**
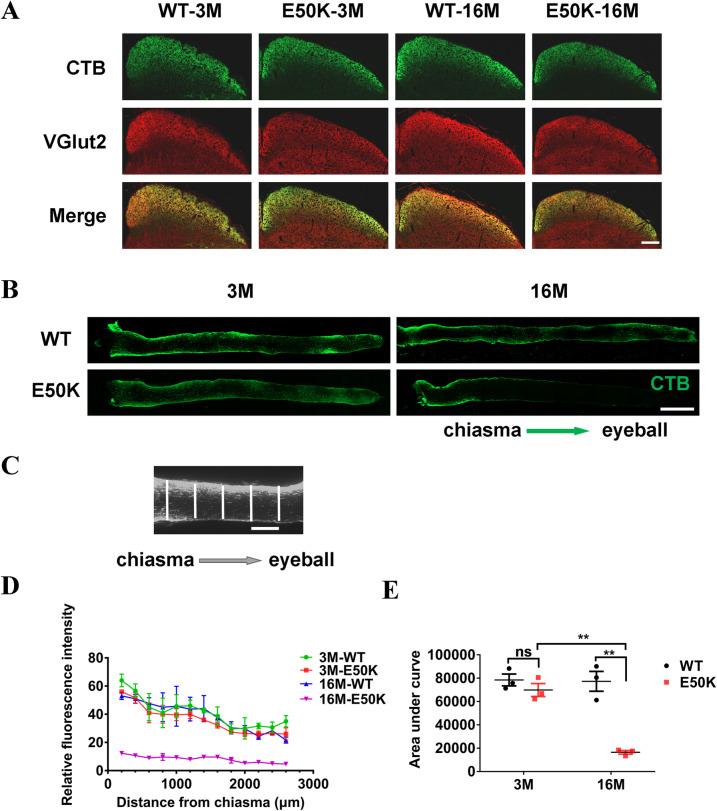
Tracing the retrograde axonal transport in WT and OPTN (E50K) mice. Frozen section of the SC was prepared 48 h after bilateral intravitreal injection of CTB-488. **A** The RGCs projection region in the SC was mapped by both CTB-488 (in green) transported from the eyes to the SC and VGluT2 (in red) expressed in SC. Scale bars, 200 μm. **B**, **C** CTB-488 was injected bilaterally into the SC. The relative fluorescence intensity across the width of the optic nerve at 100 µm intervals was quantified by ImageJ. Scale bars, 500 μm (**B**), 100 μm (**C**). **D** The relative fluorescence intensity indicated the quantity of transported CTB in optic nerves from chiasma to eyeball. **E** The area under the curve, based on graphed fluorescence intensity against distance (along the nerve), was computed using Prism (WT-3M, 78466.7 ± 5085.3, E50K-3M, 69833.3 ± 5510.3, WT-16M, 77266.7 ± 8497.9, E50K-16M, 16433.3 ± 1538.7, *n* = 3/group). (**P < 0.01).

To evaluate the retrograde axonal transport, CTB-conjugated to Alexa Fluor-488 (CTB-488) was injected bilaterally into the superior colliculus (SC), and the relative fluorescence intensity across the width of the optic nerve at 100 μm intervals was measured (Fig. 6B, C). The area under the curve was calculated by Prism 7.0, representing the accumulated fluorescence intensity from the optic chiasm to the last recording site (Fig. 6D, E). The results demonstrated that the axonal retrograde transport was significantly lower in E50K mice-16M compared to WT mice-16M (77266.7 ± 8497.9 vs. 16433.3 ± 1538.8, *P* < 0.01, *n* = 3/group). Meanwhile, the axonal transport decreased with age in E50K mice (69833.3 ± 5510.4 vs. 16433.3 ± 1538.8, *P* < 0.01, *n* = 3/group). On the contrary, in WT mice, no significant changes of axonal retrograde transport were found (78466.7 ± 5085.3 vs. 77266.7 ± 8497.9, *P* > 0.05, *n* = 3/group) (Fig. 6E). Forty-eight hours after bilateral intravitreally injection of CTB-488, 14 μm frozen sections of the SC were prepared. The CTB-488 transported from the retina to the SC mapped the RGCs projection region in SC and the RGCs axonal terminals were marked by VGluT2 (Fig. 6A). The results showed that the structure of the RGCs projection region in the SC was integrate and the terminals activity was maintained in both WT and E50K mice aged 3 months and 16 months. Thus, the decreased retrograde axonal transport in E50K mice-16M was owing to the axon itself, rather than the abnormal CTB-488 uptake at the axonal terminals.

**Fig. 7:**
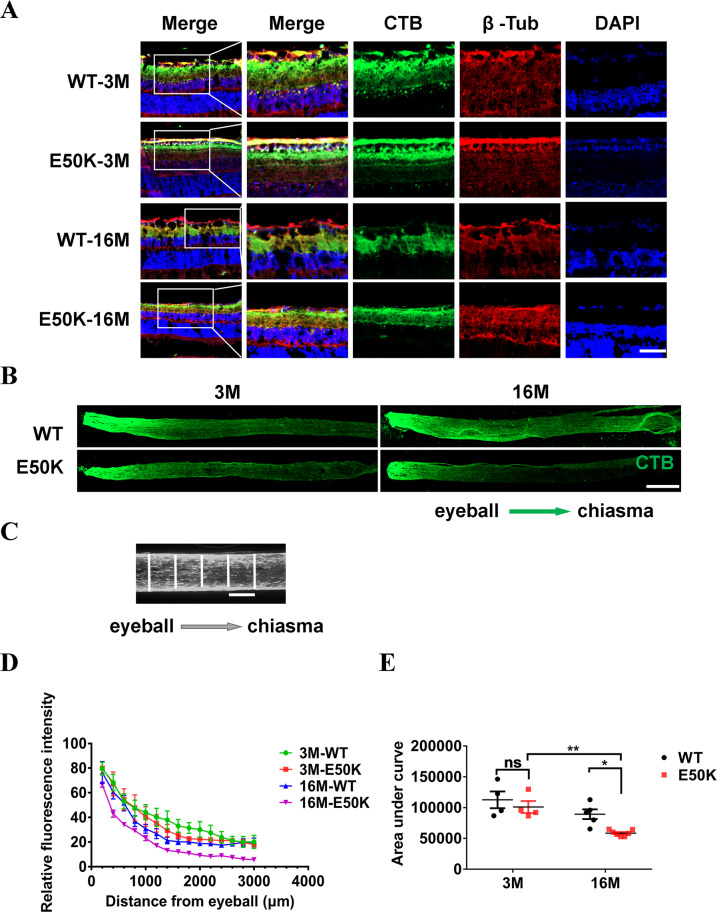
Tracing the anterograde axonal transport in WT and OPTN (E50K) mice. Frozen section of retina and optic nerve was prepared 48 h after bilateral intravitreal injection of CTB-488. **A** CTB-488 distribution in retinas and the microtubules of RGCs were marked by β-tubulin. Scale bars, 50 μm. **B**, **C** The relative fluorescence intensity across the width of the optic nerve at 100 µm intervals was quantified by ImageJ. Scale bars, 500 μm (**B**) and 100 μm (**C**). **D** The relative fluorescence intensity indicated the quantity of transported CTB in optic nerves from eyeball to chiasma. **E** The area under the curve, based on graphed fluorescence intensity against distance (along the nerve), was computed using Prism (WT-3M, 112669.5 ± 13560.3, *n* = 4, E50K-3M, 101053.0 ± 9761.1, *n* = 4, WT-16M, 89293.4 ± 7952.8, *n* = 5, E50K-16M, 58584.3 ± 1869.0, *n* = 7). (*P < 0.05, **P < 0.01).

To evaluate the anterograde axonal transport, 48 h after bilateral intravitreal injection of CTB-488, the relative fluorescence intensity across the width of the optic nerve at 100 μm intervals was measured. The analysis method is the same as that in the retrograde transportation evaluation (Fig. 7B, C). The results showed that axonal anterograde transport was significantly lower in E50K mice-16M compared to WT mice-16M (E50K-16M, 58584.3 ± 1869.0, *n* = 7 vs. WT-16M, 89293.4 ± 7952.8, *n* = 5, *P* < 0.05). Anterograde axonal transport in E50K mice decreased with age (E50K-3M, 101053.0 ± 9761.1 *n* = 4 vs. E50K-16M, 58584.3 ± 1869.0, *n* = 7, *P* < 0.01) (Fig. 7D, E). Uptake of CTB-488 at the retinal was indicated by Immunofluorescence (IF) (Fig. 7A). The RGCs microtubule were marked by β-Tubulin. The results showed that CTB-488 was distributed in the RNFL, GCL and IPL in retina of WT and E50K mice at 3 months and 16 months old, thus indicating the decreased anterograde axonal transport in E50K mice-16M did not result from lower uptake efficiency.

These suggested that the previously observed RGCs loss, retinal structural changes and visual impairment in E50K mice were at least partly due to the impaired axonal transport related to E50K mutation.

### RGCs axonal degeneration in E50K mice

**Fig. 8:**
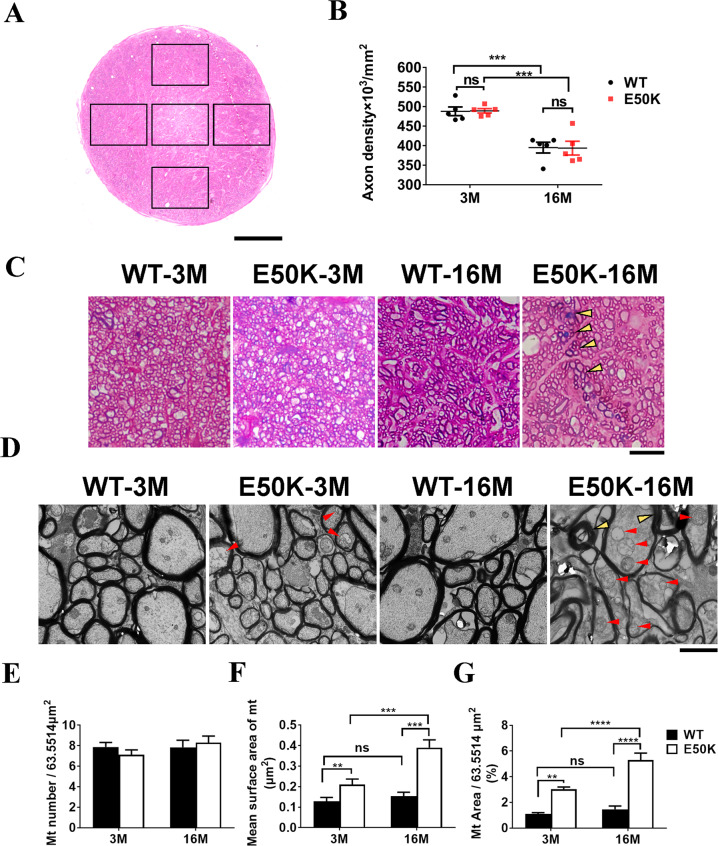
The axonal degeneration in optic nerves of OPTN (E50K) mice. **A**–**C** The semi-thin (500 nm) cross section of the optic nerve was stained with methylene-Azure II blue and basic fuchsin, then the section was visualized and photographed under a light microscope. The density of axons in each field was computed by Axon J. Scale bar in **A** indicated 50 μm. The axon density of each group was analyzed by Prism (WT-3M vs. E50K- 3M, 487.9 ± 11.2 vs. 489.2 ± 5.6, *P* > 0.05; WT-16M vs. E50K-16M, 395.0 ± 13.7 vs. 393.7 ± 17.6, *P* > 0.05, *n* = 5/group). **B** WT mice and E50K mice-3M showed normal fascicle packing and regular myelin wraps on axons in optic nerves. Disrupted axon fascicles were found in samples from E50K mice-16M (black arrowheads). Scale bar indicated 10 μm (**C**). **D**–**G** Ultra-thin sections (70 nm) were prepared in a standardized procedure for electron microscope investigation. Abnormal myelin profiles (yellow arrowheads) were found in E50K mice-16M, whereas swollen mitochondria with cristae loss (red arrowheads) were found not only in E50K mice-16M but also in E50K-3M (**D**). The mitochondrial number in single fields (*P* > 0.05) (**E**), the mean surface area of mitochondria (WT-3M, 0.11 ± 0.01, E50K-3M, 0.2 ± 0.02, WT-16M, 0.23 ± 0.01, E50K-16M, 0.31 ± 0.02) (**F**) and the proportion of mitochondria area (WT-3M, 1.09 ± 0.1, E50K-3M, 3.01 ± 0.18, WT-16M, 1.46 ± 0.26, E50K-16M, 5.29 ± 0.55) (**G**) in a single field were calculated by ImageJ to evaluated the condition of mitochondria in axons. Scale bar in **D**, 2 μm. (*n* = 5/group, **P < 0.01, ***P < 0.001, ****P < 0.0001).

The cause of axonal dysfunction might be qualitative as well as quantitative. Semi-thin sections were photographed by light microscope, and axon density of each observed area was calculated by AxonJ (Fig. 8A). No significant difference in the density of axons in the optic nerve between E50K mice and WT mice at both 3 months and 16 months old (*P* > 0.05, *n* = 5/group) (Fig. 8B). This indicated that E50K mutation does not affect the density of RGC axons. Furthermore, the E50K mice-3M showed normal fascicle packing and regular myelin wraps on axons, whereas in the E50K mice-16M, some fields exhibited disruption of axon fascicles (Fig. 8C). To ascertain whether E50K mutation led to axonal injury, the ultrastructure of axons in optic nerve were explored by electron microscopy. In morphology, swollen mitochondria with cristae loss was found in E50K mice-3M, and aggravated mitochondrial damage in E50K mice-16M. Differently, only tiny morphological changes (without statistical significance) were found in WT mice-16M, compared to WT mice-3M (Fig. 8D). The axonal mitochondrial surface area was significantly increased with no significant difference of mitochondrial number, in E50K mice compared to WT mice at both 3 months old and 16 months old (Fig. 8E–G). These results suggested that mitochondrial damage occurs prior to axonal dysfunction and RGCs loss, which may accumulate with age, gradually leading to visual impairment as outcome in E50K mice at old age.

## Discussion

NTG caused by OPTN (E50K) mutation is an autosomal dominant disease [13]. Previous studies showed that transgenic animal models with different strategies presented independent retinal pathological phenotypes and visual dysfunction [14, 15]. The expression of OPTN in transgenic animals depends on exogenous promoters, which made it difficult to accurately simulate the physiological process of OPTN in vivo. In addition, transgenic animals usually express endogenous WT OPTN protein at the same time, which to some extent compensates for the abnormal biological function caused by OPTN (E50K) mutation [14]. However, CRISPR/Cas9 gene-editing introduces single nucleotide polymorphism (SNP) mutations, which helps to accurately describe the phenotypic changes of target genes in physiological state [16]. Thus, the effect of endogenous WT OPTN protein on the physiological function of mutant OPTN (E50K) protein was minimized at gene level. Hereby, homozygous OPTN (E50K) single-site mutant mouse model, established using CRISPR/Cas9 gene-editing technology, can better simulate the pathological process caused by OPTN (E50K) gene mutation in vivo.

Number of NeuN labeled RGCs decreased in flat mount of retina in E50K mice-16M compared to WT mice-16M in our study (Fig. 5). NeuN immunoreactivity decreases in several pathological conditions such as ischemia, hypoxia and trauma [17, 18]. So this change suggested the significantly lower neuronal viability of RGCs in E50K mice. Corresponding to the above data, reduced thickness of retina and RGCs distributing layers were found through PS-OCT imaging and paraffin section H&E staining respectively in E50K mice-16M compared to WT mice-16M (Fig. 4). These changes conformed to the neurodegenerative characteristics of glaucoma. In order to exclude the effects of mechanical stress on retina or abnormal anterior chamber structure, IOP was measured by the rebound tonometer i-Care tonometry and anterior chamber structure was scanned by AS-OCT. No significant difference was found in IOP and anterior chamber structures of E50K mice aged from 3 months to 16 months compared to the WT mice, consistent with clinical findings of NTG [19]. These results implied that the visual impairment in this mouse model might be attributed to OPTN (E50K) mutation-related lower neuronal viability of RGCs rather than mechanical injury by IOP.

We further evaluated the visual function of the E50K mice through L/D-T and OMR test. L/D-T is based on the natural exploratory behavior of rodents to new environments and their aversion to brightly illuminated areas. Against the mice natural behavior, E50k mice-16M spent less time in dark compartments than WT mice-16M and E50K mice-3M in L/D-T (Fig. 2C). OMR records the movements that allow an individual to track motion of the surrounding environment. The real-time head tracking delivered quantitative OMR data, showing the number of head movements of E50K mice-16M decreased significantly than WT mice-16M (Fig. 2F). The damage of visual pathway in E50K mice-16M was further confirmed by F-VEP (Fig. 2B), which was consistent with the above results of RGSs damage. No significant visual dysfunctions were found in WT mice or E50K mice-3M according to the data of L/D-T, OMR and F-VEP. These revealed that not only RGCs damage but also visual impairment in age-related pattern was found in E50K mice, which was in accordance with the clinical aspects of NTG [7].

The mRNA level of OPTN in retinal tissue of E50K mice showed no significant difference with WT mice, which denied the influence of E50K mutation on the transcriptional level of OPTN (Fig. 3B). However, different results were found in the WB. Reduced yield of OPTN protein in E50K mice retina was detected than in WT mice at both 3 months and 16 months old (Fig. 3C, D), which should result from the lower solubility of OPTN protein in E50K mice. E50K mutation enhances the OPTN binding affinity with TANK Binding Kinase 1 (TBK1), which inhibits the self-oligomerization, leading to increased hydrophobicity [20]. This enhances the insolubility of E50K mutant OPTN, which was consistent with our observation as well. However, to our confusion, the relative level of soluble OPTN protein in the retina of E50K mice-16M was slightly higher than that of E50K mice-3M, which was consistent with our proteomic findings (data not shown). This change might be related to aging, physiological feedback or the accumulations of metabolites, but the reasons remain to be investigated with this animal model in our further work.

In order to investigate whether OPTN mutation affects the distribution of OPTN protein in the retina, relating to RGCs damage, we detected the expression of OPTN in layers of retina through immunofluorescence stained frozen section of retina. It was found that OPTN protein was expressed in nearly all retinal layers of E50K mice at 3 month and 16 month, with no significant difference from WT mice at the same month (Fig. 3A). Taken with the data of PS-OCT and paraffin section, this immunofluorescent results showed that E50K mutation did not change the overall expression level of OPTN mRNA or its protein distribution in retinal layers, but reduced retinal thickness (Fig. 4B), especially RGCs distributing layers (RNFL, GCL, and IPL) (Fig. 4D). These results prompt us to further study the mechanism of RGCs injury.

Two main roles of axonal transport are supply and clearance. Anterograde axonal transport supply the distal synapse with newly synthesized proteins and lipids to maintains axonal activity, whereas misfolded and aggregated proteins are cleared from the axon to soma by retrograde axonal transport for efficient degradation. Therefore, it is a physiological process that is critical to neuronal homeostasis and activity [12, 21–23]. Dysfunction of axonal transport is a significant pathological event leading to many adult neurodegenerative diseases (AONDs) [24]. In another study, deficiency of cargo adaptors in axon led to age-related RGCs loss [25]. These indicated the significant role of axonal dysfunction in RGCs degradation, and also prompted us to investigate the retrograde and anterograde axonal transport in E50K mice of different ages. Both decreased retrograde and anterograde axonal transport were found in E50K mice-16M but not in E50K mice-3M or WT mice (Figs. 6, and 7). The trend in our results was consistent with the data of visual impairment and RGCs loss in E50K mice (Fig. 5B). Although the mice model in this study were born with OPTN (E50K) mutation, but the visual impairments and retinal degeneration at old age of 16 months rather than at young age of 3 months (Figs. 2, 4, and 5). Therefore, as a potential pathogenic factor, age-related axonal transport disturbance was closely related to retinopathy and visual impairment in E50K mice.

As for the above series of assessment of RGCs axonal transport, CTB binds to ganglioside-monosialic acid-1 (GM1) on the surface of nerve cells like RGCs, and then was transported from cell soma to synapse via axonal transport, or the reverse route [25], indicating that tracing neurons axons through CTB-conjugated fluorescence is an effective way to assess axonal transport although this approach could be indirect when used alone. Namely when axon terminal activity gets lower, CTB uptake may be impeded, thus affecting transport evaluation. Accordingly, in this study, VGluT2 was used to mark the RGCs terminals with bio-activity in SC [26–28], and the anterograde transported CTB-488 from RGCs soma to axonal terminals was employed to map the RGCs projection region in SC (Fig. 6A). By this means, the effects of E50K mutation of OPTN on RGCs retrograde axonal transport in the mouse model were evaluated more reliably. Similar strategy was used in the assessment of anterograde axonal transport. Forty-eight hours post intravitreal injection of CTB-488, the distribution of CTB-488 in retina was evaluated together with IF method to exclude the difference in retinal uptake of CTB-488 between WT mice and E50K mice at the same age (Fig. 7A).

Intriguingly, in this study, no significant decrease of RGCs axons density was found in E50K mice-16M compared to WT mice-16M (Fig. 8B). In previous studies, accumulated mitochondrial damage is an important marker of aging and widely found in neuron axons of neurodegenerative diseases, such as Amyotrophic lateral sclerosis, Alzheimer’s disease and Huntington’s disease [29]. Inadequate ATP supply due to mitochondrial dysfunction could be responsible for defective axonal transport [30, 31]. Thus, we evaluated the changes in axonal quality of E50K mice, and by electron microscopy, the swollen mitochondria with cristae loss of RGCs axons were then found in both E50K mice-3M and E50K mice-16M (Fig. 8D). Damages in mitochondria of axons, but no detectable RGCs changes in E50K mice-3M (Figs. 5 and 8), indicating that the degeneration developed earlier in axon than in RGCs soma as a gradual process. In addition, worse mitochondrial and axonal condition in E50K mice-16M (Fig. 7E–G) indicated the axonal degeneration and mitochondrial damage is aggravated with age.

In the above results, we found significant mitochondrial damage in the degenerative axons of E50K mice-16M (Fig. 8D–G). Mitochondrial quality control via mitophagy is significant in maintaining the cellular health and is linked to several neurodegenerative diseases [29, 32, 33]. Natural OPTN protein, as an adaptor, facilitates the formation of autophagosomes [34]. However, E50K mutation leads misfolding and aggregation of OPTN protein, tending to affect the downstream bio-process like mitophagy [20, 35]. Actually, another study by our team found that OPTN (E50K) mutation inhibits autophagy [36]. These might account for the age-related accumulation of mitochondrial damage, and thus the axonal degeneration and visual impairment in E50K mice-16M in this study. In a proteomic study of our team, mitochondrial dysfunction was the top change induced by OPTN E50K mutation in retina of aged mice [37]. Optic nerve has one of the highest oxygen consumption rates and energy demands of any tissue in the body, and proper dietary supplementation could protect RGCs and reduce vulnerability to glaucoma by supporting mitochondrial health and metabolism [19, 38]. These may suggest that mitochondrial maintaining could be a potential strategy to protect RGCs axons and retina from degeneration in E50K mice.

To summary, CRISPR/Cas9-mediated genome editing was used to generate a mouse model harboring OPTN (E50K) mutation and the age-related visual dysfunction and RGCs loss of model animals was identified in this study. Then we further found that mitochondrial damage-related RGCs axonal impairment may be significant mechanism leading to the manifestations. Our study provides new insights into the pathogenesis of NTG associated with OPTN (E50K) mutation, and a new perspective as a therapeutic and intervention target for this neurodegenerative disease associated with genetic factors. As optic nerves function as a bridge between eyes and brain, therefore, axonal degeneration could affect both the viability of RGCs and the communication between RGCs and brain. In the future work, we will further study the changes in central nervous system caused by OPTN (E50K) mutation.

## Materials and methods

### Animals

Based on CRISPR/Cas9-mediated genome editing, the target genomic mutant loci was introduced to establish the mouse model of OPTN (E50K) mutation (Fig. 1A). The basic procedures are described as follows. The Cas9 mRNA and guideRNA (gRNA) were acquired through transcription in vitro. Homologous recombinant vector carrying mutation (E50K) of OPTN was established using in-fusion cloning. Zygotes of C57BL/6J mice were injected with a mixture of Cas9 mRNA, gRNA and donor vector to obtain the F0 generation of mutant mice. According to PCR and sequencing, the desired F0 generation was taken to mate with WT C57BL/6J mice to obtain heterozygote F1 generation. Then homozygous mutant mice were obtained from F1 generation hybridization, and the offspring mice were sequenced for identification. The heterozygous mice were then backcrossed with WT C57BL/6J mice to avoid the off-target effect, so as to attenuate the influence on phenotype analysis (Fig. 1B). The WT C57BL/6 mice were from the Animal Experiment Center, the Second Affiliated Hospital of Harbin Medical University. The mice were maintained in a specific pathogen free (SPF) level environment on a 12-h light/dark cycle and with free access to food and water. After appropriate anesthesia, the mice were sacrificed and tissue samples were harvested. All animal protocols are approved in advance by the Ethics Committee of the Second Affiliated Hospital of Harbin Medical University and conformed to the Guide for the Care and Use of Laboratory Animals (NIH, 8^th^ Edition, 2011) and the guidelines of the Ethics Committee of the Second Affiliated Hospital of Harbin Medical University (Permit Number: KY 2018-220).

### Western blotting (WB)

To determine the expression of OPTN protein, the retina of the mice were isolated in RIPA lysis buffer containing 1% protease inhibitors. Tissue (*n* = 6 retinas/group) is broken into homogenate by ultrasonic wave and then centrifugated at 12,000 rpm for 20 min at 4 °C. The lysates were separated by sodium dodecyl sulfate (SDS)-polyacrylamide gel electrophoresis (PAGE, 10% polyacrylamide gels), and transferred to a nitrocellulose membrane. Rabbit polyclonal anti-OPTN (1:1000, 10837-1-AP, Proteintech) was used to detect WT and E50K mutant OPTN protein. Mouse polyclonal anti-GAPDH (1:1000, 10494-1-AP, Proteintech) was used as a loading control. Goat Anti-Rabbit IgG (1:10,000, Beyotime, China) and Goat Anti-Mouse IgG (1:10,000, Beyotime, China) were used as the secondary antibodies.

### Reverse transcription-quantitative PCR (RT- qPCR)

To evaluate the transcriptional level of OPTN, the retinal tissues of mice were harvested using TRIzon Reagent (CW0580, CWBIO) at the indicated time and total RNA was extracted. Reverse transcription was performed using the First Strand cDNA Synthesis Kit (Roche, Switzerland). Quantitative detection of OPTN expression was performed with SYBGreen kit (Roche, Switzerland) reagent and Roche lightcycler 480 qPCR instrument (Roche, Switzerland). Forward primer is 5’-ATTCCGATTCACTCCTGCCC-3’, and reverse primer is 5’-GCTGGCTTATGTGGAAGGGT-3’. Fold variations between RNA samples were calculated by 2^–ΔΔct^ method after normalization to GAPDH.

### Intraocular pressure (IOP) measurement

A rebound tonometer (i-Care Technologies, Finland), specially designed for mice, was used for measurement of IOP in mice eyes according to the manufacturer’s manual. The measurement was assessed immediately after the mice were deeply anaesthetized, avoiding influence by sleep and position. The IOP of each eye was recorded by means of seven consecutive valid measurements.

### Anterior segment optical coherence tomography (AS-OCT)

The anterior chamber structures of mice were imaged through AS-OCT (Optovue, USA) under standardized dark and quite conditions. Scans were centered on the pupil and taken along the horizontal axis (nasal-temporal angles at 0° ~ 180°), using the standard anterior segment single-scan protocol (6 mm long). The parameters included ACD, ACW and AOD [39].

### Posterior segment optical coherence tomography (PS-OCT)

In order to measure the average distance from RNFL to ONL, PS-OCT (Heidelberg, Germany) were performed for imaging in vivo. After intraperitoneal injected with chloral hydrate (5%, 0.075 ml/g) for deep anesthesia, pupils were dilated with compound tropicamide eye drop (Santen, Japan), using the circular scanning mode with the diameter of 3.45 mm (system setting) and centering on optic disc to get images through a 25D lens (50744, Heidelberg, Germany). The data was computed with ImageJ software.

### Flash visual-evoked potentials (F-VEP)

Before the test, mice were kept out of the light for at least 3 h to acclimatize to the dark environment. Each test was taken in standardized dark and quiet circumstance. The mice were deeply anesthetized, and the pupil was dilated with the compound tropicamide eye drop. Then electrodes were immediately implanted subcutaneously, at occiput, mandibular part and right forelimb. Covering the contralateral vision, the reflectance waveform of the two eyes is recorded respectively.

### Light/dark transition (L/D-T) test

The apparatus used for L/D-T test consisted of a cage divided into two same chambers (403 ×328 ×414 mm), one of which was brightly illuminated (1000 lux), and the other was dark (no light). A hole (40 ×50 mm) between the two chambers allowed mice in test to pass freely. The mice for test were deprived of light for over three hours and the experiments were performed in standardized darkness and quietness. Mice were placed into the dark side and allowed to move freely for 10 min. The mice behavior in this enclosed space was captured by the camera set above and the total time spent in each side was recorded by an image software (jointly developed by Prof. Huijun Gao’s team of Harbin Institute of Technology). In between tests, the space surface was cleaned with 75% alcohol and carefully dried with wet wipes to remove the effect of olfactory [40].

### Optomotor response (OMR)

The OMR is a reflex often used to assess visual abilities based on stereotyped head movement in response to movement in the surrounding environment [41]. The experimental apparatus consists of a computer and a visual stimulus unit with a camera on top of it (Fig. 2D). A mouse was placed on a platform, and a continuous rolling raster streak was displayed on a screen when test starts (Fig. 2E). During the experiment, the camera captured video and the computer processed recognition with an automatic image-based system (jointly developed by Prof. Huijun Gao’s team of Harbin Institute of Technology). The OMR data of the mice were recorded by the algorithm of tracking head movement.

### Flat mount of retina

Flat mount of retina was performed for RGCs quantitation. Anesthetized mice were transcardially perfused with 20 ml 4% paraformaldehyde (PFA, diluted in PBS), and the eyeballs were harvested and incubated for 2 h at 4 °C. Then the retina was dissected and placed in 2% Triton X-100 (diluted in PBS) for incubation for 20 min at –80 °C and then transferred into 5% blocking buffer (normal goat serum diluted in 2% Triton X-100) for incubation for 2 h at room temperature. The retina was incubated with rabbit anti-NeuN antibody (1:50, ab177487, Abcam) diluted in 2% blocking buffer overnight at 4 °C. Then Alexa Fluor-488 conjugated goat anti-rabbit IgG (1:50, ZF-0311, ZSGB-Bio) was used as the secondary antibody at room temperature for 2 h [42]. After the retinas were mounted on object slide with vitreous body side downwards, four symmetrical radial incisions were made centered on the optic disc to flatten the retina. Coverslips were placed after antifade solution (Dako Fluorescence Mounting Medium) was applied. For evaluation of RGCs density, multiple tiled images taken with a confocal fluorescence microscope (Carl Zeiss, LSM 800, Germany) were assembled into a mosaic image that covered the entire retina flat mount. Counts were performed in a blinded fashion within a rectangular region that was 0.04 mm^2^ (200 × 200 μm) in area at 300 μm from the peripheral edge of retina using ImageJ. Eight regions were taken for qualification in each retinal sample.

### Immunofluorescence (IF)

To explore the expression and localization of OPTN protein in retina, frozen section of eyeball was harvested for IF detection [14]. The 8 μm frozen sections were blocked with 5% donkey serum before being incubated with primary antibody overnight at 4 °C and secondary antibody the next day. To visualize RGCs in green, chicken anti-NeuN (1:50, ab134014, Abcam) and goat anti-chicken IgY conjugated to Alexa Fluor-488 (1:50, ab150173, Abcam) were used as the primary antibody and the secondary antibody respectively. Furthermore, rabbit anti-OPTN (1:50, 10837-1-AP, Proteintech) as primary antibody and goat anti-rabbit IgG conjugated to Alexa Fluor 594 (1:50, ab150080, Abcam) as the secondary antibody were used to mark OPTN protein expression.

To validate the retinal uptake of Cholera toxin B (CTB), intravitreal injection of 0.5% CTB-conjugated to Alexa Fluor-488 (CTB-488, 2023245, Thermo Fisher Scientific) was performed and 8 μm frozen sections of the retina were prepared 48 h post injection. The β-Tubulin Rabbit Polyclonal Antibody (1:100, 10094-1-AP, Proteintech) was used as the primary antibody and the goat anti-rabbit IgG conjugated to Alexa Fluor 594 (1:50, ab150080, Abcam) was used as the secondary antibody.

To evaluate the structural integrity of the RGCs projection region in SC and the activity of the RGCs axonal terminal, 14 μm sections of the coronal brain frozen sections of SC were prepared 48 h after intravitreal injection of CTB-488 [28]. Recombinant rabbit anti-vesicular (VGluT2, ab216463, abcam) was used to mark RGCs terminal region in SC, and the goat anti-rabbit IgG conjugated to Alexa Fluor 594 (1:50, ab150080, Abcam) was used as the secondary antibody too.

The confocal fluorescence microscopy (LSM 800, Carl Zeiss, Germany) was employed in visualization.

### H&E staining of retinal structures

The eyeballs of the mice were surgically harvested and embedded in paraffin. The contiguous tissue on the nasal side was reserved as a marker to distinguish from the temporal side of retina. Paraffin retinal sections of 4 μm thickness, obtained from slicing along the vertical meridian through the optic nerve head, were performed with standardized process for H&E staining, and the pictures were taken with an optical microscope (Olympus, Japan). The thickness of the central (200 μm from the optic disc edge) and peripheral (200 μm from the peripheral edge of retinal) retinal tissue was measured on both nasal part and temporal part respectively with ImageJ.

### Tracing of axonal transport

Anesthetized mice were fixed in a stereotaxic frame (RWD Life Sciences, China), an incision (length 1 cm) was made to expose the skull and bregma identified as the zero point. CTB-488 (1 μl of 0.5% CTB in sterile PBS solution) was injected bilaterally into the SC. Injections were performed for not <1 min and the needle left in place for 30 s [43]. Mice were kept for 4 days after the injection and then optic nerves from chiasma to eyeball were dissected for 14 μm thick frozen lengthwise section preparation, and visualized under a confocal fluorescence microscope (Carl Zeiss, LSM 800, Germany) under the same shooting parameters and stitched into a mosaic image. The relative fluorescence intensity across the width of the optic nerve at 100 μm intervals was quantified to indicate the axonal retrograde transport of CTB-488. Then the data were plotted as a line graph against distance (along the nerve), and the area under the curve was computed using Prism 7.0 (GraphPad Software, CA, USA) [43].

In the evaluation of anterograde transport, after bilateral intravitreal injection of CTB-488 (0.5%, 1.5 μl/eye), mice were kept for 48 h and the optic nerves from eyeball to optic chiasma were prepared as discribed above [28]. Then the specimen visualization and analysis were completed according to the same process as above.

### Axonal quantification and electron microscope visualization

After the optical nerve specimens were fixed by 2% glutaraldehyde, standardized procedures were performed to prepare proximal cross-sections specimens of optic nerve [19]. Azure II is used with methylene blue and basic fuchsin for the staining of semi-thin sections (500 nm). The ultra-thin section (70 nm) was stained using osmium tetroxide (osmic acid) for observation through transmission electron microscope. Bright-field images of stained semi-thin section were captured with an inverted microscope equipped with a 100×/1.3 oil immersion objective (Olympus, Japan). With AxonJ, axon density was determined by calculating axons number in each of the five equal-sized visual fields distributed evenly at central and peripheral locations in one axonal specimen. Approximately, 60–70% of each optic nerve cross section was explored.

According to the transmission electron microscope (Hitachi, Japan) process, the ultra-thin (70 nm) sections of optic nerves were mounted on copper grids used for imaging. Two images were taken from each of five grids containing a cross section of each proximal optic nerve specimen (five nerve specimens for each group). Based on these images, the morphology, the number and surface area of mitochondria in optic nerve were explored. Using ImageJ, the area of one visual field was calculated and thus, the proportion of the total mitochondrial surface area in one visual field was obtained [19].

### Statistical analysis

Animals with poor physical condition were excluded before randomization, detection, examination and data analysis. In data collection and analysis, observers were blind to the genotype and grouping. Data were expressed as means ± SEM and were analyzed using GraphPad Prism software (Version 7.0). Two-way ANOVA followed by Tukey’s multiple comparisons test was used for analyses across multiple groups, with Student’s *t*-test used to determine significant differences between two groups. Data were presented as means ± SEM, and *P* < 0.05 was considered significant. All experiments were repeated at least three times and sample sizes were estimated from pilot experiments.

## Supplementary information

Uncropped picture of Western blotting (WB) data

Related Manuscript File

Related Manuscript File

## Data Availability

All data generated or analyzed during this study are included in this published article.
