# Supplementary figures and images for: Age-related visual impairments and retinal ganglion cells axonal degeneration in a mouse model harboring OPTN (E50K) mutation
Source: Cell Death Dis. 2022 Apr 18;13(4):362. doi: 10.1038/s41419-022-04836-3 (PMC9016082; doi:10.1038/s41419-022-04836-3)

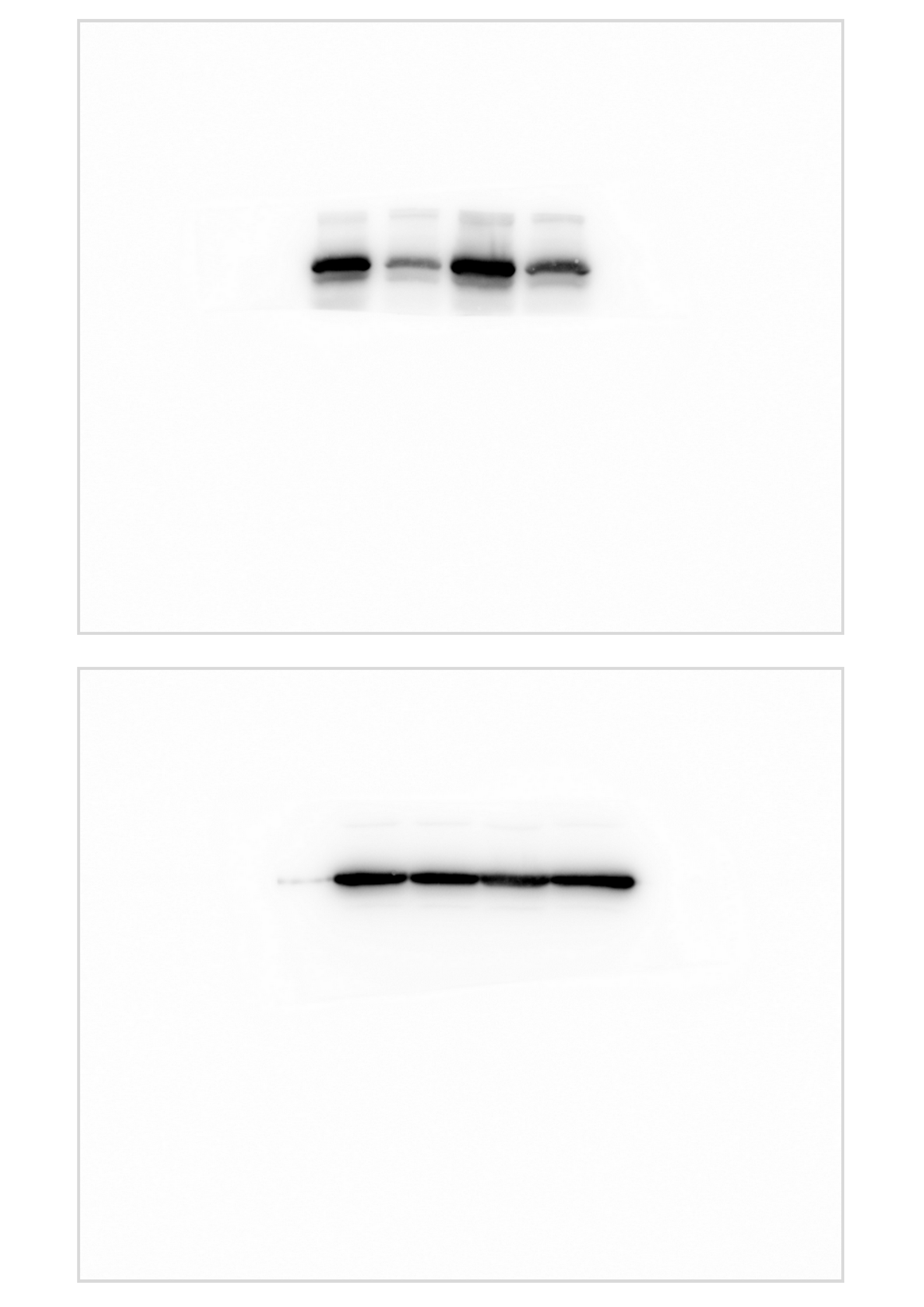

Supplement: Supplementary file 1 — Uncropped picture of Western blotting (WB) data [file 41419_2022_4836_MOESM1_ESM.tif]
