# Supplementary material for: Age-related visual impairments and retinal ganglion cells axonal degeneration in a mouse model harboring OPTN (E50K) mutation
Source: Cell Death Dis. 2022 Apr 18;13(4):362. doi: 10.1038/s41419-022-04836-3 (PMC9016082; doi:10.1038/s41419-022-04836-3)
Supplement: Supplementary file 2 — Related Manuscript File [file 41419_2022_4836_MOESM2_ESM.pdf]

DECLARATION OF CONTRIBUTIONS TO ARTICLE

ADMC

Manuscript Number:	Journal Name:	
CDDIS-21-0902R	*Cell Death & Disease*	(the 'Journal')

Proposed Title of the Contribution:

Age-related visual impairments and retinal ganglion cells axonal degeneration in a mouse model harboring OPTN (E50K) mutation (the 'Contribution)

Author(s):

Mingying Hou, Zhengbo Shao, Shiqi Zhang,Xinna Liu, Pan Fan, Menglu Jiang, Yutong Zhao, Rong Xiao, Huiping Yuan (the 'Authors')

For all *CDDis* articles, each person named as an author in the published version must be able to show he or she has contributed substantially to the article.

Authorship credit should be based on 1) substantial contributions to conception and design, acquisition of data, or analysis and interpretation of data; 2) drafting the article or revising it critically for important intellectual content; and 3) final approval of the version to be published. Authors should meet conditions 1, 2 and 3.

Any person who cannot be shown to have made a substantial contribution to the article cannot be listed as an author in the final version. The name of any person who is deemed to have made a minor contribution can, however, appear in the Acknowledgments section of the article.

Please complete the table below to indicate the contributions of all named authors to the manuscript.

Author Full Name:	Specification of Contribution to the Manuscript:
MY Hou	Conceive the idea of this study and draft the manuscript.Animal maintaining, animal experiments, data analysis and interpretation.
ZB Shao	Animal experiments protocol and design
SQ Zhang,	Animal maintaining, sequencing, animal experiments,acquisition of data
XN Liu	Animal maintaining,data analysis, acquisition of data,animal experiments
P Fan	Animal experiments,test apparatus operation instruction
ML Jiang	Animal maintaining,the data analysis, animal experiments,acquisition of data
YT Zhao	Animal maintaining, animal experiments,the data analysis
R Xiao	Animal maintaining, animal experiments
HP Yuan	Conceive the idea of this study.Revision and final approval of the version to be published.

Please complete the table below to indicate the contributions of all named authors to the figures.

Figure 1:

MY Hou, A-E,
SQ Zhang, XN Liu,C,
P Fan,D,
ML Jiang,D&E.

Figure 2:

MY Hou, A-F,
SQ Zhang, XN Liu, P Fan, A-C,
ML Jiang, D-F.

Figure 3:

MY Hou, A-D,
SQ Zhang, B,
XN Liu, C&D.

Figure 4:

MY Hou, A-D
SQ Zhang, XN Liu, P Fan, A&B,
ML Jiang, YT Zhao ,C&D.

Figure 5:

MY Hou, ZB Shao, YT Zhao and R Xiao, A&B.

Figure 6:

MY Hou, ZB Shao, SQ Zhang, XN Liu, YT Zhao and R Xiao, A&B.

Signed for and on behalf of the Author(s):	Print Name:	Date:
Huiping Yuan	Huiping Yuan	2021. 7. 23.
